# Social-emotional competencies and health-related outcomes of health promotion in upper secondary education: a scoping review of German-speaking countries

**DOI:** 10.3389/fpsyg.2026.1872950

**Published:** 2026-08-26

**Authors:** Miriam Kerzendorfer-Breithardt

**Affiliations:** Department for Educational Research, School of Education, Johannes Kepler University, Linz, Austria

**Keywords:** adolescence, DACH, health promotion, health-risk prevention, school-based interventions, social–emotional competencies, stress, upper secondary education

## Abstract

**Background:**

The implementation of health promotion and health-risk prevention programmes in educational contexts can support adolescents’ health, well-being, and psychosocial development. Social–emotional and related psychosocial competencies are key resources for coping, positive health behaviour, and risk-behaviour prevention during adolescence. However, evidence on universal programmes for upper secondary students in German-speaking countries (DACH region) that assess these competencies alongside health outcomes is scarce, and the available evidence remains insufficiently synthesised. This scoping review maps the available evidence from Germany, Switzerland, and Austria.

**Methods:**

A PRISMA-ScR–conformant scoping review searched PubPsych—including ERIC, PubMed/MEDLINE, and PSYNDEX—and the Green List Prevention registry for studies published between 2011 and November 2025. Eligible studies evaluated universal, manualised, German-language interventions targeting adolescents aged 14–19 years (grades 9–13) in the DACH region, used (cluster-)randomised controlled or quasi-experimental designs, and reported social–emotional learning–relevant outcomes. Data on programme, study, outcome, measurement, findings, and quality characteristics were extracted. Social–emotional and related psychosocial outcomes were classified using the CASEL framework. Findings were narratively synthesised.

**Results:**

Eleven studies, representing 10 programmes met the inclusion criteria, indicating a limited evidence base. Outcome coverage was uneven: programmes most often assessed self-awareness, self-management, and stress-related outcomes, whereas social awareness, responsible decision-making, broader well-being, mental health, and health-related behavioural outcomes were less consistently assessed. Reported findings were mostly modest and heterogeneous across outcome areas and study designs as well across delivery modes, school contexts, and levels of methodological quality.

**Conclusion:**

German-speaking countries have a limited and fragmented evidence base on universal upper-secondary health promotion and prevention programmes incorporating social–emotional components. Future research and practice should strengthen the assessment of social–emotional learning outcomes, improve implementation reporting, and examine how these components can be systematically integrated into programmes to promote psychosocial coping resources, well-being, and healthy lifestyles in older adolescents.

## Introduction

Health promotion programmes and health risk-related preventive interventions in educational settings are key strategies for supporting adolescents’ psychosocial functioning, well-being, and health. Educational settings provide continuous access to diverse student populations, including those with disadvantaged socio-economic or migration backgrounds, offering opportunities to reduce social and health inequities (Benningfield et al., 2015; OECD, 2023). Although universal programmes targeting entire student populations rather than only those at risk have gained attention, the available evidence base on evaluated programmes and reported outcomes for upper secondary students (14–19 years) remains limited and fragmented, partly because adolescents’ developmental transitions and the diversity of educational pathways make this age group particularly challenging to study (Jones et al., 2022). Adolescents aged 14–19 years face unique psychosocial challenges, as they encounter increasing academic and practical demands while navigating more complex social relationships and expectations for autonomy, which exacerbates vulnerability to stress, mental health problems, risk behaviours, and well-being deterioration [Health Behaviour in School-aged Children (HBSC) study, 2023; Badura et al., 2024]. These risks are further intensified by contemporary stressors such as social media pressure and the lingering effects of pandemic disruptions (Léger-Goodes et al., 2022; Garagiola et al., 2022). Structural factors compound the challenge: approximately 10% of young adults aged 19–24 years in German-speaking countries (Germany, Austria, and Switzerland, the DACH region) are not in employment, education, or training (European Commission, Directorate-General for Education, Youth, Sport and Culture, 2024; OECD, 2024).

In the DACH region, these challenges unfold within educational systems that require relatively early decisions regarding future educational pathways. Depending on the country and school structure, students are directed into academically oriented secondary schools, including grammar schools (*Gymnasien*) and vocational grammar schools, which prepare them for university entrance qualifications. Alternatively, they may enter vocational education and training (VET), which combines hands-on job experience with school-based instruction. The DACH education systems also include other secondary school types, such as comprehensive schools (e.g., *Gesamtschulen*), intermediate schools (e.g., *Realschulen*), and special-needs schools (e.g., *Förderschulen*), which differ in their curricular focus and the qualifications they provide. The choice between different educational tracks is typically made between 10 and 15 years of age and can substantially influence future education and career opportunities. Students in VET are particularly vulnerable, as reflected by their higher engagement in health-risk behaviours (e.g., substance use) compared with peers in other secondary tracks. This vulnerability may be amplified during the transition into the labour market, when new responsibilities and stressors emerge (Guertler et al., 2025).

Although individual prevention and health promotion programmes have been evaluated in these settings, an overview of interventions implemented in upper secondary education, the outcomes they report, and the educational tracks in which they have been evaluated in the DACH region is lacking. Accordingly, tailored health promotion and preventive interventions in educational settings are highly important during this formative life stage (Luo et al., 2025; Pinquart and Pfeiffer, 2020).

International meta-analytic evidence on universal prevention programmes suggests small and sometimes inconsistent effects (e.g., reductions in depression, anxiety, substance use, and conduct problems), with the corresponding benefits often declining with age (Werner-Seidler et al., 2017; Yeager et al., 2015; Onrust et al., 2016). For example, depression prevention shows moderate effects in older adolescents (> 14 years; Hedges’ *g* = 0.22), while anxiety effects decrease upon going from children (<10 years) to older adolescents (Hedges’ *g* = 0.23 to *g* = 0.12; Werner-Seidler et al., 2017). Anti-bullying programmes are effective only in early secondary school, having no reliable impact beyond grade 7 (Yeager et al., 2015). Substance use prevention exerts small but significant effects that vary with developmental stage: Smoking and alcohol outcomes improve in students in grades 10–12, whereas drug use effects remain consistently modest beyond grades (Cohen’s *d* = −0.14 to 0.07; Onrust et al., 2016). Health promotion interventions (e.g., those targeting well-being, stress management, and resilience) show promising positive effects on adolescents’ psychosocial functioning (Cilar et al., 2020; Kraag et al., 2006). Despite this evidence, many syntheses lack systematic age-specific analyses, which represents a critical research gap (Žmavc et al., 2025; Sandler et al., 2014).

Many of the above-mentioned interventions target psychosocial competencies and align with social–emotional learning (SEL), a framework introduced by the Collaborative for Academic, Social and Emotional Learning (CASEL; CASEL, 2024) that encompasses five interrelated competence areas: self-awareness, self-management, social awareness, relationship skills, and responsible decision-making (Payton et al., 2000; Committee for Children, 2023; CASEL, 2020). Drawing on emotional intelligence theory (Goleman, 1996) and a bioecological systems perspective (Bronfenbrenner and Morris, 2006), SEL represents a comprehensive educational approach that promotes these competencies through explicit instruction, student-centred learning, and coordinated classroom- and school-level practices (Domitrovich et al., 2017).

Social–emotional competencies may provide broad foundations for healthy development and successful functioning across major life domains and developmental stages (Greenberg, 2025). Their promotion is particularly relevant during adolescence, as higher levels of these competencies are associated with favourable long-term outcomes: self-efficacy, which is related to self-awareness, may facilitate school-to-work transitions (Caprara et al., 2008); empathy is related to social awareness and enhances social integration in adulthood (Allemand et al., 2015); and responsible decision-making may contribute to reducing risk behaviours, including substance use, delinquency, and early sexual activity (Parker et al., 2018). These competencies may therefore represent relevant targets for universal prevention and health promotion interventions in educational settings.

Reviews and meta-analyses indicate beneficial effects of SEL interventions on social–emotional skills, behaviour, academic outcomes, and mental health across school-age populations (Cipriano et al., 2023; Ha et al., 2025; Malti and Noam, 2016; Lee and Yoo, 2025).

For adolescents aged 14–18 years, meta-analyses reveal meaningful long-term effects, although effect sizes vary across age groups (5–10 years: *g* = 0.27; 11–13 years: *g* = 0.12; 14–18 years: *g* = 0.18; Taylor et al., 2017). However, evidence on the long-term health impact of programmes integrating SEL remains limited, particularly for programmes implemented during adolescence (Greenberg, 2025). Moreover, the interpretation and comparison of this evidence are limited because SEL-related outcomes are often measured indirectly or through related constructs, making it difficult to attribute effects to specific SEL domains (Malti and Noam, 2016).

Beyond these limitations in interpreting and comparing the evidence, questions remain regarding the cultural transferability of the US-developed CASEL framework and related programmes (Cipriano et al., 2023). International studies suggest that SEL-related programmes can be implemented across educational systems when adapted to local sociocultural and institutional conditions, including language, content, programme structure, delivery, and stakeholder involvement (Lim et al., 2026). However, the evidence base on culturally responsive SEL remains limited, heterogeneous, and predominantly US-based (Stark et al., 2021; Heidelburg et al., 2026; Hayashi et al., 2022). Even within the United States, concerns have been raised that commonly used SEL terminology, frameworks, and measures may not adequately reflect the experiences and values of culturally diverse communities (Petrokubi et al., 2019).

These considerations support the need to examine the contextual relevance of SEL-related frameworks and programmes within specific educational systems, including the DACH region (Park et al., 2025). Evidence from German-speaking educational settings provides a context-specific example of an SEL-related life-skills programme implemented and evaluated in upper secondary education. In Germany, Lions-Quest *‘Erwachsen handeln’* [Acting Responsibly], designed for students aged approximately 15–21 years in grades 9–13, was evaluated using a quasi-experimental design with a control group and follow-up (Fichtner et al., 2023). This example adds context-specific evidence from Germany to the predominantly international literature on SEL-related programmes in upper secondary education. Nevertheless, the cultural and contextual applicability of the CASEL framework across the DACH region remains insufficiently understood and, although beyond the scope of the present review, represents an important area for future research.

Despite the broader international evidence base and this context-specific example from Germany, universal health-risk prevention and health promotion programmes for students in grades 9–13 that report social–emotional or related psychosocial outcomes have not been systematically mapped in the DACH region. Existing syntheses do not adequately address these gaps, as German-language research may be overlooked when reviews include only studies in English, creating a risk of bias (Lee and Yoo, 2025). Moreover, the corresponding meta-analysis is limited to studies published up to 2010, covers a broad range of health promotion and health-risk prevention programmes rather than interventions specifically targeting upper secondary students, and does not focus on SEL-related outcomes (Beelmann et al., 2014).

To address these gaps, this scoping review maps universal prevention and health promotion interventions in upper secondary education in the DACH region. It examines health-related, social–emotional, and related psychosocial outcomes, their operationalisation, reported findings, and evidence gaps. For this purpose, the CASEL framework serves as an analytical structure for classifying conceptually related social–emotional and psychosocial outcomes.

The review is guided by three key research questions (RQs):

RQ1 Programme identification and study characteristics. Which evaluated universal health promotion and health-risk related prevention programmes implemented in upper secondary education report outcomes across health-related and social–emotional outcome areas, including related psychosocial outcomes?
RQ2 Measures and outcomes. Which outcomes across health-related and social–emotional areas, including related psychosocial outcomes, are reported, and how are they operationalised across studies?
RQ3 Reported findings and evidence gaps. What patterns of reported findings and evidence gaps emerge across programmes, outcome areas, school contexts, delivery modes, and study designs?

## Methods

The employed methodology and reporting procedures aligned with the Preferred Reporting Items for Systematic Reviews and Meta-Analyses extension for Scoping Reviews (PRISMA-ScR; Tricco et al., 2018).

### Eligibility criteria

Eligibility criteria were defined *a priori* according to the Population, Intervention, Comparison, Outcomes, Study design framework (PICOS; Thomas et al., 2024). Eligible studies involved students/adolescents aged 14–19 years or students in grades 9–13 in vocational or general education tracks. When information on age variability (e.g., standard deviation or range) was available, at least 75% of participants were required to fall within the target age group. Studies with greater variability were only included if the intervention was explicitly conducted within an upper secondary education (grades 9–13) context. Only universal interventions that were manualised (including digital formats) and delivered in German, conducted in educational contexts in the DACH region, and primarily targeted students (although teachers and parents could also participate) were included, while targeted interventions were excluded. Eligible study designs included randomised controlled trials (RCTs), cluster-RCTs, and quasi-experimental trials with a control or wait-list group. Studies had to report at least one primary or secondary outcome related to health-risk prevention (e.g., mental health) and health promotion (e.g., stress and well-being) and at least one SEL domain outcome (self-awareness, social awareness, self-management, relationship skills, responsible decision-making) assessed directly or via validated proxy measures (e.g., self-efficacy, emotion regulation, and perceived stress). Studies published between 1 January 2011 and 21 November 2025 were considered, and both peer-reviewed and grey literature (doctoral theses and reports) were included to increase the coverage of relevant evidence (Beelmann, 2021). A full description of the eligibility criteria is provided in Supplementary material 1.

### Information sources and search strategy

A systematic literature search was conducted in electronic databases and registries. PubPsych was chosen because it comprehensively covers psychological and educational research from European and German-language sources and provides access to multiple databases, including PubMed, the Education Resources Information Centre, and PSYNDEX, as well as other psychological and educational sources. The Green List Prevention (*Grüne Liste Prävention*) registry of Communities That Care was included because it specifically catalogues evidence-based prevention and health promotion programmes in German-speaking countries. The search combined programme/intervention-related terms with school-level and outcome-related terms using Boolean operators (AND, OR).

English: (Program* OR Intervention* OR Prevention* OR Training* OR Coaching) AND (‘Secondary School’ OR ‘High School’ OR ‘Vocational School’ OR ‘Vocational Education’) AND (Control* OR Eff* OR Comp*).

German: (Programm* [program*] OR Intervention*[intervention*] OR Prävention* [prevention*] OR *Vorbeugung* [prevention] OR *Training* [training] OR *Coaching* [coaching]) AND ‘*Sekundarstufe*’ [‘secondary school’] OR ‘*Gymnasium*’ [‘grammar school’] OR ‘*Realschule*’ [‘intermediate secondary school’] OR ‘*Oberschule*’ [‘secondary school’] OR ‘*Lehre*’ [‘apprenticeship’] OR ‘*Duale Ausbildung*’ [‘vocational education and training’] OR ‘*Berufsschule*’ [‘vocational school’) AND (Kontroll* [control*] OR Eff* [effect*] OR Vergleich* [comparison*] OR *Wirksamkeit* [effectiveness]).

For the Green List Prevention registry, programmes were identified using the following filter criteria.

English: Programme type: Universal AND Setting: School.

German: *Programmtyp*: *Universell* UND *Lebensumfeld*: *Schule.*

The initial search was performed in May 2025, and a second search for 2025 publications was conducted on 21 November 2025.

Following the systematic literature search, several studies were excluded because of insufficient reporting on the identified programmes. An additional supplementary targeted literature search was conducted to address these information gaps and identify further publications on the effects of the following programmes: *Effects of a Physical Education-Based Coping Training* (EPHECT), *Smart Coach* (SC), *ready4life* (R4L), *Meine Zeit ohne* (My time without, MZO). This supplementary search included citation tracking and targeted searches for programme-specific publications. In addition, one report (Lions-Quest ‘*Erwachsen handeln*’ [‘Acting Responsibly’], LQEH) was included on the basis of prior knowledge of its eligibility.

### Study selection

**Figure 1:**
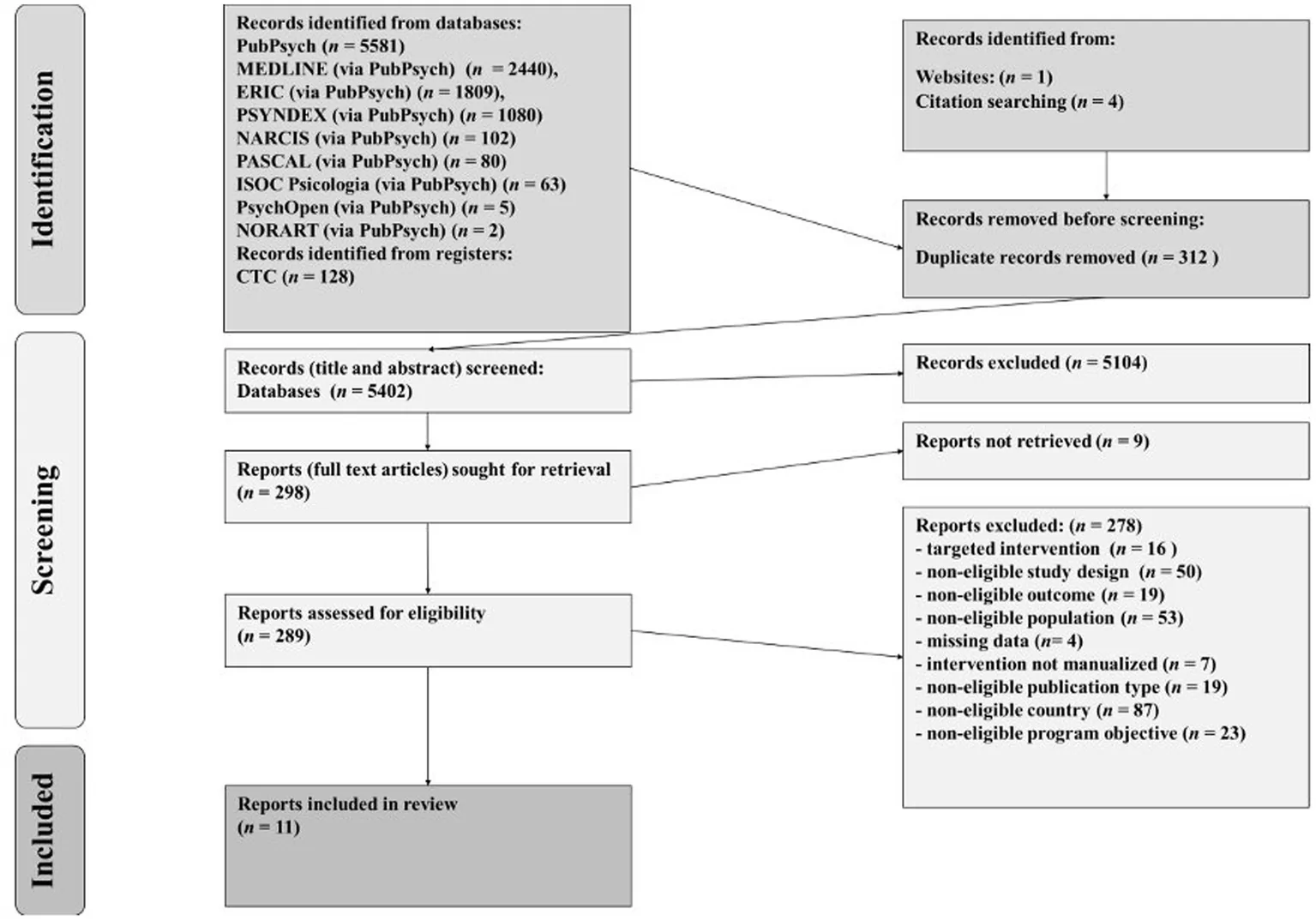
Flow diagram of the employed study selection process. Adapted from preferred reporting items for systematic reviews and meta-analyses flowchart (38).

All retrieved records were imported into Rayyan, a web-based review tool, which was used for title and abstract screening. One author conducted both title/abstract and full-text screening, using the predefined eligibility criteria. To ensure consistency, a second reviewer independently reassessed a random sample of 25% of title/abstract records, and 20% of full-text records without access to the screening decisions. Inter-rater agreement was 94% for title/abstract screening and 90% for full-text screening. Disagreements were subsequently discussed and resolved by consensus; where eligibility judgements were uncertain, methodological advice on inclusion/exclusion was sought from a second adviser. During title/abstract screening, broad exclusion reasons were recorded in Rayyan for many excluded records; however, reasons were not documented systematically for every excluded record. In accordance with standard reporting practice, reasons for excluding each study were only systematically documented during the full-text screening stage, with a reason was recorded for each excluded study (Figure 1). This study selection process was documented using a PRISMA-based flow diagram (Moher et al., 2009).

### Data extraction

Data were extracted using a standardised template developed for this review. Key extracted data are presented in the corresponding Results tables. One author extracted all relevant information from the selected studies, including study design, sample characteristics (e.g., age and educational context), intervention characteristics (e.g., format, duration, and delivery mode), comparison conditions, and reported outcomes. SEL and related psychosocial outcomes were classified according to the five CASEL competencies (CASEL, 2024) using an *a priori* coding framework. The CASEL coding framework, including operational definitions and coding rules, is provided in Supplementary material 2. All extracted data were manually checked for accuracy. When sufficient statistical information (e.g., means, standard deviations, and test statistics) was available, effect sizes (Cohen’s *d*) were calculated based on the reported data. When information was incomplete or unclear, data were extracted as reported in the original publications.

### Quality assessment

The methodological quality of the included studies was assessed using the Quality Assessment Tool for Quantitative Studies developed by the Effective Public Health Practice Project (EPHPP; Armijo-Olivo et al., 2012). This tool evaluates six domains, namely selection bias, study design, confounders, blinding, data collection methods, and withdrawals and dropouts. Each domain is rated as strong, moderate, or weak, and an overall rating is generated according to EPHPP guidelines. The quality assessment was conducted to support interpretation of the reported findings and to describe the methodological characteristics and limitations of the included evidence. It was not used as an exclusion criterion. Methodological quality ratings were visualised using the robvis R package, and the corresponding EPHPP quality assessment visualisation is provided in Supplementary material 3 (McGuinness and Higgins, 2021).

### Data synthesis

The collected data were narratively synthesised to provide an overview of the included programmes and to map their study characteristics, outcome areas, measurement approaches, reported findings, and evidence gaps.

Study characteristics, targeted outcome areas, measurement approaches, and reported findings were described. Effect sizes were extracted, where reported; where they were not reported but sufficient statistical information was available, Cohen’s d was calculated based on the reported data and presented descriptively. Regression results were also summarised, including regression coefficients and standardised coefficients (*β*), where available. To support the descriptive synthesis of reported findings, a harvest plot was created in R. Findings were categorised according to their direction and approximate magnitude based on reported results and effect sizes, supplemented by calculated effect sizes where possible.

Mixed findings were coded when findings within the same outcome area were divergent. The coding matrix used to construct the harvest plot is provided in Supplementary material 4. No meta-analysis was conducted. Missing data in primary studies were handled according to the reported methods; when missing data were not addressed, this was noted and considered in the synthesis.

## Results

### Study characteristics

Of the 5,714 records screened by title and abstract, 5,416 were excluded. Exclusion reasons were not recorded on a record-by-record basis during title and abstract screening. Based on the screening process, the main reasons were an ineligible geographical setting or target population, outcomes outside the scope of the review, such as exclusively clinical or pharmacological outcomes, and ineligible study designs, such as qualitative studies.

The remaining 298 studies underwent full-text assessment, which resulted in 278 exclusions. Nine reports were not retrieved. The most common reasons were inelegible country (*n* = 87) and ineligible population (*n* = 53). The remaining 11 studies were published between 2015 and 2025, with four (36%) and seven (64%) published after and before 2020, respectively (Figure 1).

Two studies (18%) took place in Switzerland, and nine (82%) in Germany. Nine (82%) were peer-reviewed, and two (18%) were reports. Studies covered grades 7–13 in small-, large-, and multi-school settings. Participants were aged 12–22 years (mean: 14.5–19.7 years), with the share of females ranging from 35 to 100%. The sample size varied from 29 to 4,591 students, with most studies including 50–400 participants. Follow-up rates were 62–95%, with dropout typically 16–38%. Seven studies were cluster-RCTs, and four were quasi-experimental trials. All interventions were universal, education-based, and manualised, with aims focused mainly on psychosocial resources, including stress and emotion regulation, psychosocial development, and risk prevention (Table 1). In the descriptive methodological assessment, four studies were rated methodologically weak and seven moderate (see Supplementary material 3 for the EPHPP quality assessment visualisation).

**Table 1:** Summary of included studies.

Study, intervention	Country	Study design	Comparator	Population and setting	Sample size (school/class/individual), attrition	Measurement time points	Study aim
Eppelmann et al. (2018), SBT	D	cRCT	Waitlist control, standard curriculum classes	Grade 11, grammar school (*Gymnasium*), 16.6 years, 58% female	4 schools/ NR classes /351 students (attrition T1–T2: 65/351)	T1 (baseline), post (primary)	Stress management
Fichtner et al. (2023), LQEH	D	QED	Standard curriculum education	Grades 11–13, vocational school (VET, other), grammar school (*Gymnasium*), other, 14.5 years, 49% female	6 schools/ 26 classes/ 307 students (attrition T1–T3: 125/307)	T1, post (primary), 2–3 M follow-up	SEL and civic competence
Frenkel et al. (2020), 8-sam	D	cRCT	Waitlist control	Grade 9, grammar school (*Gymnasium*), 14.6 years, 46% female	1 school/ 2 classes/ 48 students (attrition T1–T3: 7/48)	T1, post (primary), 1.5 M follow-up	Mindfulness
Gouda et al. (2016), MBSR	D	cRCT	Waitlist control	Grade 11, grammar school (*Gymnasium*), 16.2 years, 100% female	1 school/ NR classes / 29 students (attrition T1–T2: 29/29	T1, post (primary), 4 M follow-up	Health, wellbeing, and creativity
Guertler et al. (2025), R4L (digital application)	D	cRCT	Waitlist control	Vocational school (VET, grammar, other), 19.7 years, 46% female	5 schools/ 376 classes/ 2,568 students (attrition T1–T3: 1326/2568)	T1, post (primary), 6 + 12 M follow-up	Addictive behaviour reduction
Haug et al. (2021), SC (digital application)	CH	cRCT	Assessment-only control	Secondary school/upper secondary school (not specified), 15.4 years, 55% female	NR schools/ 89 classes/ 1,473 students (attrition T1–T2: 240/1473)	T1, post (after 6 M baseline), 18 M follow-up	Addictive behaviour reduction
Institut für Therapie- und Gesundheitsforschung IFT-Nord, gemeinnützige GmbH (2023), MZO (digital application)	D	cRCT	Waitlist control	Vocational school (not specified), 19.2 years, 45% female	17 schools/ 277 classes/ 4,591 students (attrition: 1730/4591)	T1, post (after 2 M baseline)	Short-term addictive behaviour reduction
Lang et al. (2016), EPHECT	CH	cRCT	Standard curriculum education	Vocational school (VET), 16.2 years, 35% female	1 school/ 8 classes/ 131 students (attrition: 19/131)	T1, post, 6 M follow-up	Stress management
Luong et al. (2019), MBSR	D	QED	Waitlist control	Grade 11, grammar school (*Gymnasium*), 16 years, 78% female	3 schools / nr classes/ 81 students (attrition T1–T2: 8/81)	T1, post (after 3 M baseline, for IG vs. CG comparison), 4 + 7 M follow-up (only IG)	Mental health, SEL, and creativity
Schiepe-Tiska and Bertrams (2015), SG	D	QED	Standard curriculum education	Vocational school (not specified), 16.04 years, 63% female	2 schools / 6 classes /106 students (attrition: NR)	T1, post (primary)	Well-being, self-esteem, and self-efficacy
Vasileva et al. (2019), Jobfit	D	QED	Waitlist control	Grades 7–12, secondary school (*Realschule*), vocational school, special education school (*Förderschule*), 15.7 years, 38% female	20 schools / nr classes / 393 students (attrition T1–T2: 118/393)	T1, post (primary, for IG vs. CG comparison), 3 + 6 M follow-up (only IG)	Work and social behaviour

CH, Switzerland; cRCT, cluster-randomised controlled trial; CG, control group. D: Germany, EPHECT, *effects of a physical education based coping training*; IG, intervention group; Jobfit, *Jobfit-Training*; LQEH, Lions-Quest ‘*Erwachsen handeln*’; *8-sam* (mindfulness/self-regulation); MBSR, mindfulness-based stress reduction; M, month; MZO, *Meine Zeit ohne*; nr, not reported; QED, quasi-experimental design; R4L, ready4life; SBT, *Stressbewältigungstraining* (stress management training); SC, *Smart Coach*; SG, *Schulfach Glück* (school subject happiness programme); VET, vocational education and training.

### RQ1: programme identification and intervention characteristics

Ten interventions were identified across the included studies: *Stressbewältigungstraining* [stress management training] (SBT; Eppelmann et al., 2018), *Lions-Quest ‘Erwachsen handeln’* [Acting Responsibly] (LQEH; Fichtner et al., 2023), *8-sam* [mindfulness/self-regulation programme] (Frenkel et al., 2020), *Mindfulness-Based Stress Reduction* (MBSR; Gouda et al., 2016; Luong et al., 2019), *Smart Coach* (SC; Guertler et al., 2025), *ready4life* (R4L; Haug et al., 2021), *Effects of a Physical Education-Based Coping Training* (EPHECT; Lang et al., 2016), *Meine Zeit ohne* [My Time Without] (MZO; Institut für Therapie- und Gesundheitsforschung IFT-Nord, gemeinnützige GmbH, 2023), *Schulfach Glück* [school subject happiness] (SG; Schiepe-Tiska and Bertrams, 2015), and *JobFit-Training* (JobFit; Vasileva et al., 2019).

School type. Interventions were mainly implemented in vocational schools (e.g.*, Berufsschulen*; 36%) and grammar schools *(Gymnasien*; 36%), with three programmes (27%) targeting mixed school types.

Delivery format. Most interventions (65%) were delivered as extracurricular programmes, and the rest (35%) were curriculum-based or embedded within existing subjects. Three interventions (27%) used exclusively digital formats.

Intervention content. All digital programmes (27%) focused on addictive behaviours and life-skills promotion. In contrast, classroom-based interventions (73%) addressed broader health promotion and well-being outcomes. These programmes primarily targeted SEL-related competencies such as problem-solving, social skills, emotion regulation, or self-efficacy and emphasised mindfulness, stress management, and lifestyle topics, showing a stronger emphasis on holistic well-being rather than risk reduction.

Facilitators. Classroom-based programmes were delivered mostly by teachers (40%) or external facilitators (30%), while digital interventions (30%) were largely self-guided, with one including an introductory facilitator session.

Theoretical frameworks. The 10 interventions were based on diverse theoretical frameworks, including cognitive-behavioural therapy, mindfulness-based stress reduction (MBSR), and social-cognitive and health behaviour models, positive psychology, SEL, yoga, and experiential learning.

Programme duration. Classroom-based programmes ranged from multi-session formats to longer embedded programmes spanning a school year. Digital interventions were self-guided or bi-weekly sessions, while embedded formats integrated content into existing school subjects (Table 2).

**Table 2:** Description of included interventions.

Study	Intervention name and aim	Target group and delivery setting	Theoretical basis/approach	Intervention content	Duration/frequency/format	Facilitator
Eppelmann et al. (2018)	Stress management training: Stress coping	Grammar school, extracurricular	Cognitive behavioural therapy	Relaxation, group and performance coping, cognitive restructuring, future planning	3 × 90 min PE, ≥2 weeks apart, classroom-based	External facilitator
Fichtner et al. (2023)	LQEH: Unspecific prevention programme for life skill building	Grammar school/vocational school, 15–21 years, curriculum-based/embedded	Competence-based socialisation approaches, salutogenic models	Group work, self-efficacy, problem solving, human rights, social skills, civic participation	Five modules over 10 months, units/frequency not reported (COVID 19), classroom-based	Trained teacher
Frenkel et al. *(*2020)	8-sam: Mindfulness-based programme for self-regulation strengthening	Grammar school, extracurricular	MBSR	Mindfulness, sensory awareness, thoughts and emotions, stress coping, experiential exercises	8 × 25 min sessions/4 weeks, classroom-based	External facilitator
Gouda et al. (2016); Luong et al. (2019)	Mindfulness training: Stress coping and well-being enhancement	Grammar school (students and teachers), extracurricular	MBSR	Mindfulness, sensory awareness, psychoeducational inputs, experimental and practical experiences	8 × 120 min/8 weeks + one full-day training, classroom-based	External facilitator
Guertler et al. (2025)	R4L: Digital app for reducing addictive behaviours and boosting life skills	Vocational school, extracurricular	Social cognitive theory, health action process approach	Self-management skills, social skills, substance use resistance skills	2 × 6 modules (alcohol, tobacco, cannabis, social media/gaming, stress, social skills), 8 × 60 min/8 weeks, digital	Digital
Haug et al. (2021)	SC: Digital app for reducing addictive behaviours and boosting life skills	Secondary/grammar/vocational school, extracurricular	Social cognitive theory, health action process approach	Self-management skills, social skills, substance use resistance skills	60 min intro + 6 months, 2–4 texts/week, self/social/substance skills, digital	External facilitator/ Digital
Institut für Therapie- und Gesundheitsforschung IFT-Nord, gemeinnützige GmbH (2023)	MZO: Digital app for reducing addictive behaviours	Vocational school, extracurricular	Social learning theory, self-efficacy theory	Voluntary two-week reduction/abstinence from substances or behaviours	Daily app, 2 weeks, 2 × notifications, digital	Digital
Lang et al. (2016)	EPHECT: Coping skill promotion and perceived stress reduction	Vocational school, embedded	Experiential learning theory, cognitive-transactional stress model	Stress knowledge, emotion- and problem-focused coping, relaxation, applied consolidation in PE classes	20 min per week/12 weeks, classroom-based	Teacher
Schiepe-Tiska and Bertrams (2015)	SC: Promotion of well-being and self-efficacy	Secondary school/grammar school/ vocational school (grades 8–12), embedded	Positive psychology, social learning theory, self-efficacy theory, wellbeing	Self-exploration, wellbeing and self-efficacy, five modules (life joy, achievement, nutrition, movement, body expression), experiential and practical activities	40 × 120 min/school year, classroom-based	Teacher
Vasileva et al. (2019)	Jobfit: School-to-work transition support via social–emotional skills and work behaviour	All school types, ages 13–20 years, extracurricular	Cognitive behavioural therapy, SEL model	Goal-setting, future planning, self-regulation, social–emotional skills, empathy, assertiveness, role-play, self-monitoring	10 × 90 min/10 weeks, classroom-based	Teacher

MBSR, mindfulness-based stress reduction; SEL, social and emotional learning; PE, physical education.

### RQ2: measures and outcomes

Outcome areas and measurement approaches. To address RQ2, reported outcomes were grouped into SEL-related psychosocial outcomes/constructs and health-related outcomes. SEL-related psychosocial outcomes were classified according to the five CASEL competencies using an *a priori* coding framework. Further details on coding rules, measurement instruments, and operationalisation are provided in Supplementary material 2 (CASEL coding framework) and Supplementary material 4 (full measure description), as well as in Tables 3, 4.

**Table 3:** Mapping of social–emotional, related psychosocial, and health-related outcomes across CASEL domains by study.

Study/intervention	SAw	SM	SoA	RS	RDM	SEL-related psychosocial outcomes / constructs mapped to CASEL domains	Health-related outcomes	Measures
Eppelmann et al. (2018), SBT		■				Knowledge of stress and coping; coping strategies	Stress levels; symptom burden; quality of life	CASQ; PQ; YSR; KIDSCREEN
Fichtner et al. (2023), LQEH	■		■	■		Self-efficacy; empathy/religious diversity; conflict resolution/critique skills; expression of opinions and feelings; communication; emotional competence	nr	Study-specific items – not reported
Frenkel et al. (2020), 8-sam	■/▧	▧				Observing internal experiences; accepting without judgement; self-noticed mind wandering; attention performance; externally noticed mind wandering	Perceived stress	KIMS-short; d2; PSQ
Gouda et al. (2016), MBSR	■/▧	■		▧		Mindfulness; school-related self-efficacy; general self-efficacy; self-regulation; emotional competences; interpersonal problems	Perceived stress; anxiety; depression; test anxiety; openness to experience; creativity; engagement	FMI; SES; SRS; ERSQ; IIP; PSQ; HADS
Guertler et al. (2025), R4L				■		Social competence	Stress symptoms; problematic internet use; cigarette smoking; alcohol consumption; cannabis use	AI; single-item stress measure; short compulsive internet use scale; cigarette consumption; AUDIT-C; quantity–frequency index; lifetime and past-six-months frequency
Haug et al. (2021), SC				■		Interpersonal competences	Alcohol consumption; cigarette smoking; cannabis use days; perceived stress; perceived well-being; problem drinking; tobacco smoking; cannabis use	ICQ-10; AUDIT-C; cigarettes/month; cannabis use days/30 days; Swiss Juvenile Study stress item; WHO-5; HBSC 30-day prevalence items
Institut für Therapie- und Gesundheitsforschung IFT-Nord, gemeinnützige GmbH (2023), MZO	■				▧	General self-efficacy; any behaviour change	Addictive behaviours: social media use, video gaming, alcohol consumption, gambling, e-product use, cigarette smoking, cannabis use; positive mental well-being; physical activity	ASKU; BFAS; IGD-SF; 30-day prevalence items; ≥50% reduction items; PMH-9; self-reported days/month of physical activity; GAHBI
Lang et al. (2016), EPHECT		■				Adaptive coping; maladaptive coping	Perceived stress	SVF-KJ; ASC
Luong et al. (2019), MBSR	■/▧	■		▧		Mindfulness; school-related self-efficacy; general self-efficacy; self-regulation; emotional competences; interpersonal problems	Perceived stress; anxiety; depression	FMI; SES-S; SES-G; SRS; ERSQ; IIP; PSQ; HADS-A; HADS-D
Schiepe-Tiska and Bertrams (2015), SG	■	▧				Self-esteem; self-efficacy expectations; emotional stability	Positive affect; negative affect; affect balance; life satisfaction; general happiness; extraversion	Rosenberg; SES-G; SPANE; SWLS; Subjective Happiness Scale; BFI
Vasileva et al. (2019), Jobfit	■	■	▧	■		Calmness/self-esteem; learning behaviour; reliability/foresight; social behaviour	nr	IBES; SSL/LSL

SAw, self-awareness; SM, self-management; SoA, social awareness; RS, relationship skills; RDM, responsible decision-making. ■, direct mapping to CASEL domain; ▧, indirect or proxy mapping to CASEL domain; ■/▧, both direct and indirect/proxy indicators were identified for this domain. Blank, domain not assessed; nr, not reported or not applicable. Measure abbreviations are reported as used in the original studies; full names of all measures are provided in the Supplementary material 4.

**Table 4:** Overview of intervention effects reported in the included studies.

Author, study	Measurement type	SEL domain/SEL-related outcomes an*d* measures, effect sizes and significance	Health-relate*d* outcomes and measures, effect sizes and significance
Eppelmann et al. (2018), (SBT)	Self-report (students)	SM: Knowledge gain (Knowledge about stress and coping): *β* = 0.60, *p* < 0.001; SM: Coping strategies (PQ, CASQ): Active β = 0.00, *p* = 0.965; Internal β = −0.06, *p* = 0.535	Stress levels (*P*Q): *d* ≈ 0.06, *p* = 0.439; Symptom burden (YSR): *d* ≈ 0.03, *p* = 0.757; Quality of life (KIDSCREEN-10): *d* ≈ 0.04, *p* = 0.666;
Fichtner et al. (2023), (LQEH)	Self-report (students)	RS: Conflict resolution / critique skills (nr): *d* ≈ −0.24, *p* = 0.084; SAw: Self-efficacy – initiative (nr): *d* ≈ 0.04, *p* = 0.087; SoA: Em*p*athy / religious diversity (nr): *d* ≈ 0.23, *p* = 0.095	nr
Frenkel et al. *(*2020), (8-sam)	Self-report (students)	SM: Attention performance (*d*2): *d* ≈ −0.03, *p* = nr; SAw: Observing internal experiences (KIMS-short): *d* ≈ −0.48, *p* = nr; SAw: Accepting without judgement (KIMS-short): *d* ≈ −0.23, *p* = nr; SM: Externally noticed MW (ex*p*erimenter probe): *d* ≈ −0.02, *p* = nr; SAw: Self-notice*d* MW (self-re*p*ort): *d* ≈ −0.31, *p* = nr;	Perceived stress (*P*SQ) (T2): *d* ≈ −0.26, *p* = nr
Gouda et al. (2016), Mindfulness-Based Stress Reduction (MBSR)	Self-report (students)	SAw: Mindfulness (FMI): *d* ≈ 0.53, *p* = 0.065; SAw: School self-efficacy (SES-S): *d* ≈ 0.62, *p* = 0.014; SAw: General self-efficacy (SES-G): *d* ≈ 0.48, *p* = 0.091; SM: Self-regulation (SRS): *d* ≈ 0.66, *p* = 0.018; SM: Emotional competences (ERSQ): *d* ≈ 0.47, *p* = 0.079;	Stress (PSQ): *d* ≈ −0.67, *p* = 0.047; Anxiety (HADS-A): *d* ≈ −0.66, *p* = 0.075; Depression (HADS-D): *d* ≈ −0.32, *p* = 0.251; Test anxiety – emotionality (TAI-E): *d* ≈ −0.43, *p* = 0.082; Test anxiety – worrying (TAI-W): *d* ≈ 0.11, *p* = 0.698; Interpersonal problems (IIP): d ≈ −0.68, *p* = 0.005; Openness (NEO-O): *d* ≈ 0.14, *p* = 0.479; Creativity (TCT-DP): *d* ≈ 0.57, *p* = 0.116; Engagement (UWES): *d* ≈ 0.35, *p* = 0.167
Guertler et al. *(*2025), (R4L)	Self-report (students)	RS: Social competence (AI): *d*6m = 0.10, *d*12m = 0.19, *p* = 0.002	Stress (PSQ): *d*6m = 0.10, *d*12m = 0.20, *p* < 0.001; Problematic internet use (Short Compulsive Internet Use Scale): *b* = 0.058, *p* = 0.004; Cigarette consumption: *b* = 0.031; *F* = −, *p* = 0.007; Alcohol consumption (AUDIT-C, quantity-frequency index): *b* = 0.011; *p* = 0.145; Cannabis use (lifetime and past-six-month frequency): *b* = 0.039; *p* = 0.078
Haug et al. (2021), (SC)	Self-report (students)	RS: Social skills (ICQ-10): CC *d* = 0.08, *p* = 0.10; ITT *d* = 0.08, *p* = 0.07	Alcohol quantity (AU*D*IT-C, amount): CC *d* = −0.05, *p* = 0.06; ITT *d* = −0.08, *p* = 0.03; Cigarette quantity (cigarettes/month): CC *d* = −0.11, *p* = 0.07; ITT *d* = −0.13, *p* = 0.01; Cannabis use days (*d*ays/30): CC *d* = −0.15, *p* = 0.02; ITT *d* = −0.12, *p* = 0.053; Perceived stress (1–5 swiss Juv. Stu*d*y): CC *d* = −0.13, *p* = 0.03; ITT *d* = −0.15, *p* = 0.02; Well-being (WHO-5): CC *d* = 0.05, *p* = 0.24; ITT *d* = 0.07, *p* = 0.16; Problem drinking (AUDIT-C, 30-day prevalence): CC OR = 0.64, *p* = 0.01; ITT OR = 0.71, *p* = 0.07; Tobacco smoking (30-day prevalence): CC OR = 0.82, *p* = 0.48; ITT OR = 0.83, *p* = 0.46; Cannabis use (30-day prevalence): CC OR = 0.90, *p* = 0.60; ITT OR = 1.24, *p* = 0.21;
Lang et al. (2016), (Ephect)	Self-report (students)	SM: Adaptive coping (SVF-KJ): post-intervention; Time × Group *F*(1, 111) = 5.86, *p* < 0.05, η^2^ = 0.050; path-model direct intervention effect β = 0.28, *p* < 0.001; stable at follow-up (ns); SM: Maladaptive coping (SVF-KJ): post-intervention (Time × Group ns); overall time effect pre–post, *F*(1, 111) = 7.92, *p* < 0.01, η^2^ = 0.067; stable at follow-up (ns);	Stress perception (ASC): post-intervention (ns); at 6-month follow-up (Time × Group), *F*(1, 111) = 3.82, *p* < 0.05, η^2^ = 0.035
Luong et al. (2019), (MBSR)	Self-report (students)	SAw: Mindfulness (FMI): *p* = 0.05, *d* = 0.34; SM: Self-regulation (SRS): *p* = 0.03, *d* = 0.33; SM: Emotional competencies (ERSQ): *p* = 0.01, *d* = 0.47; SAw: School-specific self-efficacy (SES-S): *p* = 0.13, *d* = 0.21; SAw: General self-efficacy (SES-G): *p* = 0.06, *d* = 0.30; RS: Interpersonal competencies (IIP): *p* = 0.17, *d* = 0.20;	Perceived stress (PSQ): overall *p* = 0.01, *d* = 0.46; Anxiety (HADS-A): *p* = 0.005, *d* = 0.52; Depression (HADS-D): *p* = 0.04, *d* = 0.36; Openness to experience (NEO-O): ns; *F*(1, 70) = 1.00, *p* = 0.32, η^2^ₚ = 0.014; Drawing creativity (TCT-DP): ns; *F*(1, 70) = 0.58, *p* = 0.45, ηₚ^2^ = 0.008; Verbal creativity (VCT): *F*(1, 70) = 0.60, *p* = 0.44, ηₚ^2^ = 0.015, *d* = 0.22
Institut für Therapie- und Gesundheitsforschung IFT-Nord, gemeinnützige GmbH (2023) (MZO)	Self-report (students)	RDM: Any behaviour change (GAHBI): 73.0% vs. 68.8%; OR = 1.24, 95% CI 1.05–1.46, *p* = 0.010; Saw: General self-efficacy (ASKU): ns, OR = 1.05, 95% CI 0.90–1.22, *p* = 0.553;	Social media (BFAS): 48.4% vs. 41.8%; OR = 1.31, 95% CI [1.13, 1.53]; *p* < 0.001; Video games (IGD-SF): 26.8% vs. 25.2%; OR = 1.09, 95% CI [0.91, 1.31]; ns; Alcohol (30-day prevalence): 8.6% vs. 8.0%; OR = 1.12, 95% CI [0.86, 1.44]; *p* = 0.402; Gambling (30-day prevalence): 6.7% vs. 6.0%; OR = 1.14, 95% CI [0.81, 1.61]; *p* = 0.447; E-products (≥50% reduction): 9.9% vs. 9.3%; OR = 1.12, 95% CI [0.85, 1.47]; *p* = 0.453; Cigarettes (≥50% reduction): 10.9% vs. 10.0%; OR = 1.08, 95% CI [0.73, 1.59]; *p* = 0.300; Cannabis (30-day prevalence): 4.5% vs. 4.1%; OR = 1.12, 95% CI [0.86, 1.44]; *p* = 0.407; Positive mental well-being (PMH-9): ns; OR = 1.15, 95% CI [0.99, 1.34]; *p* = 0.068; Physical activity (days/month): ns; OR = 1.05, 95% CI [0.86, 1.27]; *p* = 0.640.
Schiepe-Tiska and Bertrams (2015), (SG)	Self-report (students)	SAw: Self-esteem (Rosenberg): *F*(1, 104) = 6.59, *p* = 0.01, η^2^ = 0.06; SAw: Self-efficacy expectations (G-SES): ns; *F*(1, 104) = 1.20, *p* = 0.28, η^2^ = 0.01;	Positive affect (SPANE): stable; *F*(1, 104) = 6.35, *p* = 0.01, η^2^ = 0.06; Negative affect (SPANE): stable; *F*(1, 104) = 5.52, *p* = 0.02, η^2^ = 0.05; Affect balance (SPANE): stable; *F*(1, 104) = 8.65, *p* < 0.001, η^2^ = 0.08; Life satisfaction (SWLS): ns; *F*(1, 104) = 1.06, *p* = 0.31, η^2^ = 0.01; General happiness (Subjective Happiness Scale): stable; *F*(1, 104) = 3.60, *p* = 0.06, η^2^ = 0.03; Emotional stability – life satisfaction (BFI): *F*(1, 102) = 6.74, *p* = 0.01; Emotional stability – general happiness (BFI): *F*(1, 102) = 3.36, *p* = 0.07; Emotional stability – self-esteem (BFI): *F*(1, 102) = 3.76, *p* = 0.05; Extraversion (BFI): ns
Vasileva et al. (2019), (Jobfit)	Self-report (students), Observer-rated (teacher)	RS: Social behaviour (SSL/LSL): *p*ost-intervention; *F*(1, 267) = 4.27, *p* = 0.037, η^2^ = 0.02; SM: Learning behaviour (SSL/LSL): stable *p*ost-intervention; Time × Grou*p* interaction η^2^ = 0.02; SAw: Calmness/self-esteem (IBES): *p*ost-intervention; *F*(1, 267) = 8.77, *p* = 0.003, η^2^ = 0.03; SM: Reliability/foresight (IBES): *p*ost-intervention; *F*(1, 267) = 8.37, *p* = 0.004, η^2^ = 0.03.	not evaluated

Only intervention effects relevant to the review objectives are presented. Subgroup, moderator, and other differential-effect analyses were outside the scope of the review and are therefore not reported. Where possible, reported effect estimates were converted to Cohen’s *d* to facilitate comparisons across studies; converted values are indicated by the symbol ≈. CC, complete-case analysis; CI, confidence interval; ITT, intention-to-treat analysis; MW, mind-wandering; nr, not reported; ns, not statistically significant; OR, odds ratio; RDM, responsible decision-making; RS, relationship skills; SAw, self-awareness; SM, self-management; SoA, social awareness; *b*, unstandardised regression coefficient; *d*, Cohen’s *d*; *F*, *F* statistic; *p*, probability value; β, regression coefficient; η^2^, eta squared; ηp^2^, partial eta squared. Measure abbreviations are reported as used in the original studies; the full names of all measures are provided in Supplementary material 4 (full measure descriptions).

Across studies, the outcome landscape was concentrated around intrapersonal domains. Self-awareness—operationalised through school –related and general self-efficacy (SES-S and SES-G), self-esteem (Rosenberg), calmness/self-esteem, and mindfulness (FMI, KIMS-short)—was the most frequently represented CASEL domain (7/11, 64%) across 11 studies. Self-management (6/11, 55%) included coping (SVF-KJ, CASQ), knowledge of stress and coping, self-regulation (SRS), emotional competencies/emotion regulation (ERSQ), and attention performance (d2, d2-R, KL/BZO).

Relationship skills (5/11, 45%) were operationalised via social competence (AI), interpersonal competencies (ICQ-10), social behaviour (SSL/LSL), conflict resolution/critique skills, communication, and interpersonal problems (IIP). Social awareness (2/11, 18%) was directly mapped in one study through empathy/religious diversity and indirectly mapped in another through social behaviour.

Responsible decision-making (1/11, 9%) was identified only as an indirect/proxy domain and was operationalised through a global ‘any behaviour change’ indicator (GAHBI), alongside behavioural outcomes related to digital media use, substance use, gambling, and physical activity.

Health-related outcomes and measures. Perceived stress—operationalised through the Perceived Stress Questionnaire (PSQ), single-item stress measures, the Swiss Juvenile Study stress item, and stress perception (ASC) – was the most frequently reported health-related outcome (8/11, 73%). This indicates variability in both standardised and study-specific assessment approaches.

Well-being, affect, and life satisfaction (4/11, 36%) were assessed via WHO-5, SPANE, SWLS, the Subjective Happiness Scale, and PMH-9. Anxiety and depression (3/11, 27%) were measured using HADS-A/D, while test anxiety (1/11, 9%) was operationalised using TAI-E/W.

Quality of life (KIDSCREEN-10; 1/11, 9%) and symptom burden (YSR; 1/11, 9%) each appeared in one study. Health behaviour outcomes were mainly assessed in programmes targeting addictive behaviour or risk reduction. These outcomes included substance use (3/11, 27%), digital/media use (2/11, 18%), gambling, cigarette smoking, e-product use, cannabis use, and physical activity. They were operationalised through AUDIT-C, quantity-frequency indices, the short Compulsive Internet Use Scale, BFAS, IGD-SF, 30-day prevalence items, ≥50% reduction indicators, HBSC items, and self-reported days per month of physical activity. Other psychosocial outcomes, including personality/engagement (2/11, 18%), measured via BFI/NEO-O, UWES, and creativity (2/11, 18%), operationalised through TCT-DP, VCT, were reported less consistently.

Overall, the studies used a highly heterogeneous set of measures, with similar constructs such as self-efficacy, coping, stress, and well-being operationalised using different instruments across studies. The coverage of SEL-related outcomes was uneven, with a greater emphasis on intrapersonal domains, particularly self-awareness, and self-management, than on social awareness or responsible decision-making. Health-related outcomes were concentrated around stress-related measures, whereas broader mental health and health behaviour outcomes were assessed less consistently (Tables 3, 4 and Supplementary material 2 [CASEL coding framework] and 4 [full measure descriptions]).

### RQ3: reported findings and evidence gaps

**Figure 2:**
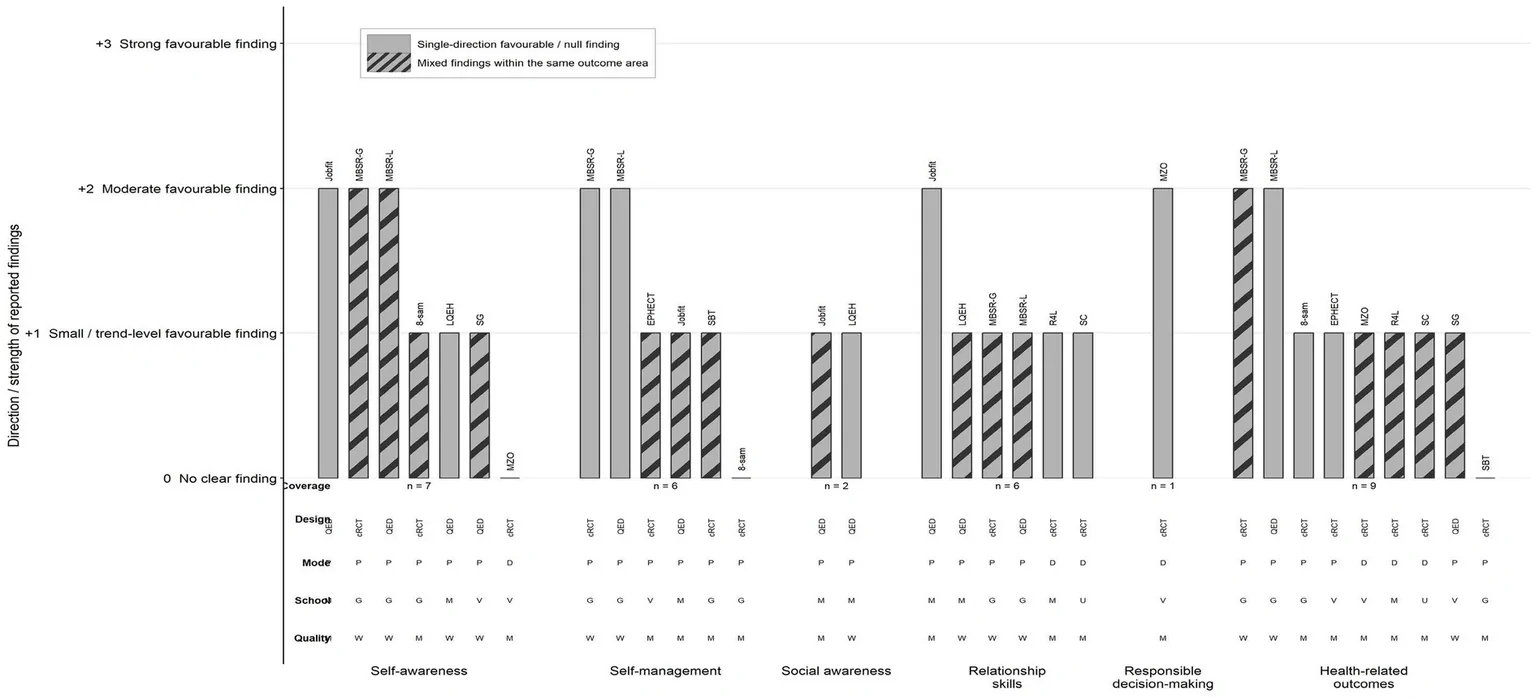
Harvest plot of reported findings across outcome areas. Bars represent studies within outcome areas. Bar height shows the coded descriptive finding from no clear effect to strong favourable findings; it does not represent an effect size. Hatched bars indicate mixed findings within the same outcome area. Coverage (n) indicates the number of studies per outcome area. Categories were based on reported or calculated effect sizes where available and otherwise on statistical tests or authors' descriptions; detailed coding is provided in Supplementary material 5 (harvest plot coding matrix) and Table 4. Programme/study labels: SBT, *Stressbewältigungstraining* (Eppelmann et al., 2018); LQEH, *Lions-Quest ‘Erwachsen handeln’* (Fichtner et al., 2023); 8-SAM, *8-sam* (Frenkel et al., 2020); MBSR-G, *Mindfulness-Based Stress Reduction* (Gouda et al., 2016); R4L, *ready4life* (Guertler et al., 2025); SC, *Smart Coach* (Haug et al., 2021); EPHECT, *Effects of a Physical Education-Based Coping Training* (Lang et al., 2016); MBSR-L, *mindfulness-based stress reduction* (Luong et al., 2019); MZO, *Meine Zeit ohne* (Institut für Therapie- und Gesundheitsforschung IFT-Nord, gemeinnützige GmbH, 2023); SG, *Schulfach Glück* (Schiepe-Tiska and Bertrams, 2015); Jobfit, *JobFit* (Vasileva et al., 2019). Design: cRCT, cluster-randomised controlled trial; QED, quasi-experimental design; Mode: P, in-person; D, digital school; G, grammar school; V, vocational school; M, mixed school types; U, upper-secondary/secondary school not further specified. Quality: study quality (EPHPP), M, moderate; W, weak.

Reported findings were descriptively synthesised across programme types, outcome areas, study designs, delivery modes, school contexts, and methodological quality. Because the included studies differed substantially in outcome constructs, measurement instruments, effect metrics, follow-up periods, and analytic approaches, the findings are presented as patterns of reported effects rather than as comparative effectiveness estimates. An overview of reported, and where possible, calculated effect sizes is provided in Table 4. Figure 2 presents a descriptive harvest plot for reported findings summarising categorised reported findings across outcome areas.

Patterns across CASEL domains and related psychosocial outcome areas. The most consistent favourable findings were reported across *self-awareness* and s*elf-management.* Self- awareness-related outcomes included mindfulness, self-efficacy, self-esteem, calmness/self-esteem, and other related intrapersonal constructs. Mindfulness-based programmes reported favourable findings for mindfulness and self-efficacy, although some mindfulness subdomains showed mixed or null findings, indicating that effects differed depending on the specific facet assessed. Self-management outcomes were mainly operationalised through coping, self-regulation, emotion regulation, stress-related knowledge, and attention. Findings in this domain were generally favourable, particularly for coping, self-regulation, and emotion regulation, but were not consistent across all measures or programmes.

Findings for relationship skills were more heterogeneous but showed some favourable patterns. Reported outcomes included social competence, interpersonal competencies, social behaviour, conflict resolution/critique skills, communication, and interpersonal problems. Favourable findings were reported in both in-person and digital programmes, although the assessed constructs differed considerably across studies. In contrast, social awareness and responsible decision-making were only sparsely represented. Social awareness was reported in a small number of studies, mainly through empathy/religious diversity, or social behaviour. Responsible decision-making was represented indirectly through behaviour-change indicators in one digital prevention programme. This indicates a clear evidence gap in the assessment of these two CASEL domains.

Patterns across health-related outcomes. Across health-related outcomes, the broadest and most consistent coverage concerned stress-related outcomes. Stress was assessed through perceived stress, stress symptoms, or study-specific stress indicators. Favourable findings were reported particularly in mindfulness-based and stress-management programmes. Mental-health outcomes, including anxiety and depressive symptoms, were assessed less frequently but showed favourable findings in mindfulness-based interventions. Well-being-related outcomes, including affect, affect balance, life satisfaction, general happiness, emotional stability-related indicators, and positive mental well-being, showed more mixed patterns. Some favourable findings were reported for affective and emotional-stability-related indicators, whereas broader outcomes such as life satisfaction and general happiness showed no clear change. Health-risk behaviour outcomes were mainly addressed in digital prevention programmes. Reported findings suggested favourable findings for some targeted behaviours, particularly digital media use, e-product use, cigarette consumption, and specific behaviour-change indicators. However, broader prevalence indicators for alcohol, gambling, cannabis use, and physical activity often showed no clear change.

Patterns across programme type and delivery mode. Patterns also differed by programme type and delivery mode. In-person programmes mainly addressed broader SEL-related and psychosocial outcomes, including mindfulness, coping, self-regulation, self-efficacy, stress management, and social behaviour. Digital programmes were more strongly focused on health-risk prevention, particularly addictive behaviour and lifestyle-related outcomes. Both delivery modes showed favourable reported findings in selected areas, but they targeted different outcome profiles, limiting direct comparison.

Patterns across school context, study design, and methodological quality. School contexts were heterogeneous. Grammar-school studies were more often linked to mindfulness, stress-management, well-being, and self-awareness-related outcomes, whereas vocational or mixed-school settings more often included health-risk prevention, coping, behaviour change, or work and social behaviour outcomes. However, school type was not always clearly reported, and programme content differed substantially across settings. Therefore, school-type patterns should be interpreted descriptively rather than as evidence of differential effectiveness.

Most studies used cluster-randomised or quasi-experimental designs, and methodological quality was rated as moderate or weak. Favourable findings were reported across study designs, but their interpretation was limited by heterogeneous outcome measures, small samples in some studies, attrition, variable follow-up periods, and incomplete reporting. Follow-up evidence was inconsistent, and long-term effects were rarely assessed systematically. The heterogeneity of interventions, outcome operationalisations, and statistical reporting prevented quantitative pooling.

Evidence gaps. Overall, the reviewed programmes reported mostly moderate to small favourable findings across selected SEL-related psychosocial and health-related outcomes, particularly self-awareness, self-management, stress-related outcomes, and targeted health-risk behaviours. However, the evidence base remains fragmented. Key gaps include uneven CASEL-domain coverage, limited assessment of social awareness and responsible decision-making, heterogeneous measurement approaches, limited long-term follow-up, moderate or weak methodological quality, and restricted comparability across delivery modes, school types, and study designs (Figure 2; Table 4).

## Discussion

This scoping review synthesises evidence on universal, manualised health promotion and prevention programmes targeting adolescents in upper secondary education (grades 9–13, mean age 14–19 years) in the DACH region (2011–2025), with a focus on health-related, and social–emotional outcomes, including related psychosocial outcomes. This developmental stage is particularly relevant because adolescents are consolidating self-regulatory capacities, identity-related self-concepts, peer and social competencies, and coping strategies while facing increasing academic, vocational, and autonomy-related demands. Overall, the findings indicate a limited, fragmented evidence base —namely, the uneven coverage of SEL-related domains, heterogeneous measurement approaches, and restricted comparability across programme types, school contexts, and study designs.

### Limited evidence on universal programmes for upper secondary students in the DACH region

Only 11 of the 5,714 initially identified studies met the inclusion criteria, with only 10 programmes identified. This reflects the scarcity of health promotion and prevention programmes with social–emotional components for this age group. This gap is concerning given the importance of psychosocial support during this demanding developmental stage, which may influence long-term psychosocial well-being (Jones et al., 2022). International research also reports fewer SEL interventions for older adolescents (Cipriano et al., 2023), and the lack of evaluations in the DACH region (particularly in Austria and Switzerland), underscores a regional research gap and the need for programme development and evaluation.

Most interventions were embedded within broader health or risk prevention frameworks rather than explicitly targeting SEL. This may reflect the locally limited dissemination of the CASEL framework, with many interventions grounded in related theoretical traditions—such as cognitive-behavioural approaches, life-skills education, health behaviour models, mindfulness, or experiential learning. Nevertheless, many programmes addressed constructs closely aligned with SEL and are relevant to adolescents’ social–emotional development.

### Uneven coverage and effects across SEL domains

Given the limited dissemination of the CASEL framework in German-speaking contexts, most programmes did not explicitly conceptualise their objectives as SEL and relied mostly on related psychosocial proxy measures such as mindfulness, coping, or self-esteem. This reflects a broader international trend, as validated SEL measures remain scarce, particularly for adolescents (Ross and Tolan, 2018). Such proxy measures are useful because they capture psychologically relevant mechanisms, but they also limit comparability across studies and may underrepresent SEL as a multidimensional construct.

A central pattern was the uneven coverage of SEL domains, in line with international evidence (van de Sande et al., 2019). Interventions mainly targeted intrapersonal domains related to self-awareness and self-management, whereas social awareness and responsible decision-making were rarely represented. This imbalance may be relevant because responsible decision-making supports adolescents in reflecting on their values, anticipating consequences, and making thoughtful choices (Moore and Gregory, 2024). Higher decision-making competence in late adolescence has been associated with more adaptive coping and favourable behavioural outcomes (Parker et al., 2018).

The limited representation of social awareness may likewise be important, as longitudinal evidence suggests continuity in empathy-related and prosocial capacities from adolescence into early adulthood and links these capacities to prosocial behaviour and interpersonal functioning (Li et al., 2025; Allemand et al., 2015). Together, these findings indicate an important gap in the coverage of key dimensions of adolescents’ social–emotional development.

### Heterogeneous health outcomes dominated by stress-related measures

Stress-related outcomes were the most frequently measured health-related outcomes across the included studies. This suggests that stress represents a relevant concern in adolescence and that corresponding mitigation strategies are highly pertinent. These findings align with international evidence indicating that universal SEL programmes yield modest but meaningful improvements in psychosocial functioning in adolescence (Durlak et al., 2011).

At the same time, broader mental-health, well-being, and health behaviour outcomes were assessed less consistently. Findings for well-being-related outcomes appeared particularly mixed, and broader indicators such as life satisfaction or general happiness often showed no clear change. Health-risk behaviour outcomes were mainly addressed in digital prevention programmes, with more favourable findings for specific targeted behaviours than for broader prevalence indicators. Overall, health-related findings varied considerably across outcome areas and programme types.

### Digital and in-person programmes targeted different outcome profiles

The findings suggest that delivery mode was closely linked to intervention focus. In-person programmes mainly addressed broader SEL-related and psychosocial outcomes, including mindfulness, coping, self-regulation, self-efficacy, stress management, and social behaviour. Digital programmes were strongly focused on health-risk prevention, especially substance use and lifestyle-related outcomes. As the two delivery modes addressed different intervention targets and outcome profiles, direct comparisons of their effectiveness were not possible.

Across both delivery modes, reported findings were generally favourable but often modest or inconsistent, and their interpretation was limited by heterogeneous intervention designs, outcomes, follow-up periods, and study quality. International reviews show a similar pattern: digital interventions for adolescents have been applied across mental-health, health-promotion, and risk-prevention targets, with generally favourable feasibility and acceptability but mixed or modest effectiveness findings. Digital mental-health interventions appear particularly promising for anxiety and depressive symptoms, whereas evidence for other outcomes, including alcohol use, remains less consistent (Magwood et al., 2024; Badawy and Kuhns, 2017).

International evidence additionally suggests that digitally guided interventions may achieve better adherence and lower dropout than fully automated approaches, while their transferability across educational and social contexts remains insufficiently understood. Evidence is therefore still limited, both internationally and in the DACH region, regarding which programmes are most suitable for digital, in-person, or blended delivery in upper secondary education. This represents an important research gap, as school types may differ in their student populations, organisational structures, and institutional conditions (Magwood et al., 2024; Lehtimaki et al., 2021).

### Educational contexts, study design, and methodological quality limited comparability

Educational settings emerged as a relevant but difficult-to-interpret dimension. Grammar-school studies were often linked to mindfulness, stress management, well-being, and self-awareness-related outcomes. In contrast, vocational or mixed-school settings more often included health-risk prevention, coping, behaviour change, or work and social behaviour outcomes. However, programme content, delivery format, and outcome selection differed substantially across settings. School-type patterns should therefore be interpreted descriptively rather than as evidence of differential effectiveness.

This is particularly relevant in the DACH region, where educational pathways are strongly structured by school type and vocational education plays a major role, including specific psychosocial demands linked to school-to-work transitions. However, inconsistent reporting and limited analysis of school type restrict conclusions about differences between educational settings.

Included studies used cluster-randomised or quasi-experimental designs, and methodological quality was rated as moderate or weak. Although favourable findings were reported across study designs, interpretation was limited by heterogeneous measures, variable follow-up periods, attrition, small samples in some studies, and incomplete reporting. The evidence should therefore be understood as a descriptive map of reported findings rather than as a robust comparative estimate of programme effectiveness. Together, these findings indicate a fragmented but psychologically relevant evidence base for universal promotion and prevention in late adolescence, with implications for research, practice, and policy.

### Researchers

Given the importance of psychosocial coping resources for adolescents’ health, well-being, and developmental adjustment, future health promotion and prevention programmes should more systematically incorporate social–emotional components and evaluate related outcomes. This gap is internationally relevant, as evidence on programmes combining health promotion, risk prevention, and social–emotional learning for older adolescents remains limited beyond the DACH region.

New programmes incorporating social–emotional components for adolescents aged 14–19 years should address the full range of CASEL domains more systematically. Current programmes focus mainly on intrapersonal competencies, while social awareness and responsible decision-making were underrepresented in the current evidence base. In addition, age-appropriate SEL instruments should be developed and validated to enable comparisons across studies and educational contexts.

Future evaluations should improve reporting of outcome measures, effect estimates, intervention fidelity, dosage, and implementation context, particularly in vocational and mixed-school settings in the DACH region. Addressing these aspects would further contribute to closing international gaps in implementation research on school-based health promotion and prevention.

Finally, future interventions should be age-appropriate and tailored to adolescents’ developmental needs and motivations, as these have been identified as key characteristics of effective programmes (Yeager et al., 2015).

### Practitioners

Although this review focused on German-speaking countries, the findings are relevant to other education systems. Given the heterogeneity of educational settings, programmes should be selected according to students’ needs and backgrounds, educational pathways, and developmental stage. From a health-psychological perspective, schools and other educational contexts are important settings for strengthening psychosocial coping resources, stress regulation, well-being, and health-related behaviour. Therefore, SEL-related principles should be embedded not only in specific programmes but also in everyday teaching practices, classroom and school climate, and wider school life (CASEL, 2020; Oberle et al., 2016).

### Policymakers

At the policy level, SEL-related health promotion should be more systematically embedded in educational and public-health strategies. International and European frameworks increasingly emphasise well-being, key competencies, lifelong learning, and inclusive education. However, older adolescents in upper secondary and vocational pathways may still receive insufficient attention, particularly in evaluated programmes. Stronger integration into frameworks such as Education and Training 2030 (Council of the European Union, 2021) could support more coherent implementation and evaluation. For example, SEL-related outcomes could be incorporated into existing school health monitoring frameworks, such as the Health Behaviour in School-aged Children study, to enable systematic assessment of psychosocial competencies alongside health indicators. In addition, SEL-informed health promotion modules could be embedded in initial and continuing teacher education curricula.

Given adolescents’ high psychosocial needs, limited psychosocial support for many young people, and the limited evidence base for upper secondary education, SEL-related health promotion should be understood as part of broader psychosocial support for this age group.

National governments should therefore mandate the implementation and evaluation of SEL-related health promotion, provide teacher training, and establish cross-sectoral collaboration between education, public health, youth services, and vocational training systems. Long-term funding and coordinated governance are essential to ensure sustainable support, implementation, evaluation, and public-health benefits.

### Strengths and limitations

The strengths of this scoping review include its focus on upper secondary education, a developmental stage that has received little attention in SEL-related health promotion research. The clearly defined age range and regional focus enable a nuanced understanding of intervention availability and characteristics in German-speaking contexts. The review applies a rigorous search strategy, including only empirically evaluated manualised universal programmes with randomised or quasi-experimental designs to ensure methodological rigour. By including both peer-reviewed and grey literature, the review also reduces the risk of publication bias and captures evidence that may be particularly relevant in educational and public-health contexts.

A comprehensive perspective on intervention design and delivery is provided by integrating outcomes on social–emotional competencies, health indicators, and key implementation characteristics (e.g., educational setting and delivery mode).

Several limitations should be noted. The small number of included studies (*n* = 11) and their methodological and conceptual heterogeneity precluded meta-analytic synthesis, limiting statistical generalisability. Restricting eligibility to randomised or quasi-experimental designs excluded other study types that could have provided additional insights, for example into implementation, acceptability, delivery modes, programme characteristics, contextual adaptation, and the range of outcomes examined.

Only universal programmes are considered, excluding targeted interventions for at-risk groups. Moreover, SEL-related outcomes were often measured indirectly through proxy constructs, and the mapping to CASEL domains required interpretative coding. Although an *a priori* framework was used, some classifications therefore remained partly judgement-based. A further limitation is the use of the CASEL framework for outcome classification. Developed primarily in the US context, it may not fully capture the diverse theoretical foundations underpinning DACH programmes, including cognitive-behavioural, mindfulness-based, experiential learning, salutogenic and positive psychology approaches (Eppelmann et al., 2018; Fichtner et al., 2023; Gouda et al., 2016; Luong et al., 2019; Schiepe-Tiska and Bertrams, 2015; Vasileva et al., 2019). Nevertheless, CASEL provided a pragmatic and consistent framework for classifying and synthesising outcomes across heterogeneous interventions and study designs.

Furthermore, implementation factors—such as dosage, fidelity, and contextual adaptation—were insufficiently examined, which is a key limitation and an important research gap for future studies aiming to identify the characteristics of effective interventions. Despite these limitations, the review offers important insights into current research on adolescent school health promotion. Overall, the review highlights the potential relevance of universal school-based interventions during late adolescence, while showing that current evidence remains fragmented and provides only limited insight into psychological mechanisms underlying social–emotional development, health behaviour, and well-being.

## Data Availability

The original contributions presented in the study are included in the article/Supplementary material, further inquiries can be directed to the corresponding author.
