# Supplementary material for: Social-emotional competencies and health-related outcomes of health promotion in upper secondary education: a scoping review of German-speaking countries
Source: Front Psychol. 2026 Aug 26;17:1872950. doi: 10.3389/fpsyg.2026.1872950 (PMC13553867; doi:10.3389/fpsyg.2026.1872950)
Supplement: Supplementary file 1 [file Table_1.DOCX]

**Supplementary File 1.** Eligibility criteria for study selection

| **Domain** | **Inclusion Criteria** | **Exclusion Criteria** |
| --- | --- | --- |
| **Population** | *Students in upper secondary school;* grades 9-13 (academic/vocational); studies conducted in Germany, Switzerland and Austria; participants aged - 14–19 years (≥75% within range, broader age ranges included only if situated in upper secondary context) | Students not in grades 9-13, predominantly younger than 14 or older than 19 years |
| **Intervention** | *Universal, structured health promotion and prevention programmes; manualbased* (including digital formats); primary participants: students  (teachers/parents may also participate); delivered in German; primary aim: health -risk prevention/health promotion | Targeted programmes; non structured or curriculum-based only; interventions not conducted in upper secondary school (grade 913); non- (psychosocial), health related, or purely academic/ educational programs without a prevention or health promotion focus |
| **Comparison** | *Waitlist or control group* | Irrelevant or non-comparable Interventions |
| **Outcome** | Included: ≥1 health outcome (e.g., wellbeing, stress, mental health, social skills/behavior, health behaviors) AND ≥1 SEL Domain outcome (self-/social awareness, self-management, relationship skills, responsible decision making; measured directly or via validated proxy measures (e.g. self-efficacy, perceived stress) | No measurement health related outcomes, or SEL-outcomes; -non-validated or unspecific  outcome measures |
| **Study Design** | Quantitative analysis required; minimum: pre–post measurement; eligible study types: RCTs, clustercontrolled trials, and quasi-experimental studies with a control/waitlist group | Cross sectional or purely correlational studies; purely qualitative studies; case studies or single-group designs without comparison; descriptive reports or programme descriptions without evaluation |
| **Publication Year** | Published between 1 January 2011 and 22 November 2025 | Published before January 2011 and 22 November 2025 |
| **Language** | English and German | Articles in other languages |
| **Publication Type** | Peer-reviewed journal articles; final reports (e.g. of funded projects); grey literature (e.g. research reports, conference proceedings); dissertations | Opinion papers, commentaries, editorials; purely theoretical or conceptual papers without empirical evaluation; non-scientific publications (e.g. magazines, press articles) |

**Supplementary File 2. Coding framework for social and emotional learning (SEL) outcomes aligned with the CASEL framework**

*Outcomes were coded according to their primary conceptual focus. Where constructs overlapped conceptually, coding decisions followed the boundary rules specified in the final column.*

| **SEL dimension** | **Definition aligned with the CASEL framework** | **Coding rule/ boundary** | **Example of outcomes** |
| --- | --- | --- | --- |
| **Self-awareness** | The ability to understand one’s own emotions, thoughts, and values and how they influence behavior. This includes accurately assessing one’s strengths and limitations, with a well-grounded sense of confidence and purpose. | Code outcomes that reflect awareness, identification, or evaluate the self. Do not include outcomes focused on regulation of emotions or behavior; these should be coded as self-management | self-concept, self-efficacy, emotional self-awareness, identity development |
| **Self-management** | The ability to regulate one’s emotions, thoughts and behaviors effectively in different situations. This includes managing stress, controlling impulses, sustaining motivation, and working toward personal and academic goals. | Code outcomes that reflect regulation, control, or management of internal states or behavior. If the primary focus is on self-perception or self-evaluation, code as self-awareness. | emotion regulation, stress management, self-discipline, self-motivation, planning and organizational skills |
| **Social awareness** | The ability to take the perspective of and empathize with others, including those from diverse backgrounds and cultures, and to understand social and ethical norms for behavior. | Code outcomes that reflect understanding other’s emotions, perspectives, or social contexts. If the primary focus is on interactional behavior or maintaining relationships, code as relationship skills. | empathy, perspective-taking, compassion, respect for others, recognition of social norms and supports |
| **Relationship skills** | The ability to establish and maintain healthy and rewarding relationships with diverse individuals and groups through effective communication, cooperation, and conflict resolution. | Code outcomes that reflect interpersonal behavior, communication, or relationship management. If outcome primarily reflects understanding other than interacting with them, code as social awareness. | communication skills, positive relationship building, teamwork, conflict resolution, help-seeking, help-offering |
| **Responsible decision-making** | The ability to make caring and constructive choices about personal behavior and social interactions based on ethical standards, safety concerns, and the evaluation of consequences for self and others. | Code outcomes that reflect judgement, choice-making, or evaluation of consequences. If the outcome primarily reflects behavioral or emotional regulation rather than decision processes, code as self-management. | problem-solving, moral reasoning, judgement and decision-making, critical thinking, evaluation of consequences, responsible behavior |

**Note**. Definitions are based on the framework of the Collaborative for Academic, Social, and Emotional Learning (CASEL) (20).

**Supplementary File 3.** Risk-of-bias assessment of the included studies presented as a traffic-light plot, created with robvis.

**Note**. Own illustration based on the included studies; created with robvis (44).

**Supplementary File 4.** Overview of measurement instruments used in the included studies

| **Measurement** | **Full name** | **Instrument source** | **Included study/studies** |
| --- | --- | --- | --- |
| AI | Assertion inventory | Gambril, Richery 1975 | Guertler *et al.* 2025 |
| ASC | Adolescent stress questionnaire | Byrne, Davenport, Mazanov 2007 | Lang *et al*. 2016 |
| AUDIT-C | Alcohol use disorders identification test – consumption | Rumpf *et al.* 2013,  Bush *et al*. 1998 | Guertler *et al.* 2025, IFT Nord 2023, Haug *et al*. 2021 |
| BFAS | Bergen facebook addiction scale | Andreassen *et al.* 2012 | IFT Nord 2023 |
| BFI | Big five inventory | Rammstedt, John 2005 | Schiepe-Tiska *et al.* 2015 |
| CASQ | Coping across situations questionnaire | Seiffge-Krenke 1995 | Eppelmann *et al*. 2018 |
| SVF-KJ | Coping questionnaire for children and adolescents | Hampel, Petermann, Dickow 2001 | Lang *et al.* 2016 |
| ERSQ | Emotion regulation skills questionnaire | Berking, Znoj 2008 | Gouda *et al.* 2016, Luong et al. 2019 |
| FMI | Freiburg mindfulness inventory | Walach *et al.* 2006 | Gouda *et al.* 2016, Luong *et al.* 2019 |
| SES-G | General self-efficacy scale | Schwarzer, Jerusalem 1999 | Gouda *et al*. 2016; Luong et al. 2019; Schiepe-Tiska et al. 2015 |
| ASKU | General self-efficacy short scale  *(Allgemeine Selbstwirksamkeit Kurzskala)* | Beierlein *et al.* 2013 | IFT Nord 2023, Luong et al. 2019 |
| HADS-A | Hospital anxiety and depression scale – anxiety | Hermann-Lingen *et al.* 2005 | Gouda *et al.* 2016, Luong et al. 2019 |
| HADS-D | Hospital anxiety and depression scale – depression | Hermann-Lingen *et al.* 2005 | Gouda *et al.* 2016, Luong et al. 2019 |
| Cannabis use (HSBC) | Cannabis use preceding 30 days, 1 item from the Health behavior in school-aged children, German version | Marmet *et al.* 2015 | Haug *et al*. 2021 |
| IGD-SF | Internet gaming disorder scale – short form | Pontes, Griffiths 2015 | IFT Nord 2023 |
| ICQ-10 | Interpersonal competence questionnaire (short form) | *Coroiu et al.* 2015 | Haug *et al.* 2021 |
| IIP | Inventory of interpersonal problems, German version | Horowitz *et al.* 2000 | Gouda *et al.* 2016, Luong *et al.* 2019 |
| IBES | Inventory of work-related characteristics and self-assessments, *(Inventar zu berufsbezogenen Eigenschaften und Selbsteinschätzungen)* | Marcus 2006 | Vasileva *et al.* 2019 |
| KIMS-short | Kentucky inventory of mindfulness skills, German version | Ströhle *et al*. 2010 | Frenkel et al. 2020 |
| KIDSCREEN-10 | Kidscreen-10 index | Ravens-Sieberer *et al.* 2010 | Eppelmann *et al*. 2018 |
| NEO-O | Openness to experience scale, NEO-FFI (NEO Five-factor inventory, German version) | Borkenau, Ostendorf 1993 | Luong et al. 2019 |
| PSQ | Perceived stress questionnaire, German version | Fliege *et al.* 2001, 2005 | Frenkel *et al.* 2020, Gouda *et al*. 2016, Luong *et al*. 2019 |
| PMH-9 | Positive mental health scale | Lukat *et al.* 2016 | IFT Nord 2023 |
| PQ | Problem questionnaire | Seiffge-Krenke 1995 | Eppelmann *et al*. 2018 |
| Rosenberg | Rosenberg self-esteem scale | Collani, Herzberg 2003 | Schiepe-Tiska *et al.* 2015 |
| SWLS | Satisfaction with life scale | Diener *et al.* 1985 | Schiepe-Tiska *et al*. 2015 |
| SPANE | Scale of positive and negative experience | Diener *et al*. 2010 | Schiepe-Tiska *et al.* 2015 |
| SES-G | General self-efficacy scale | Jerusalem, Schwarzer 1999 | Schiepe-Tiska *et al.* 2016 |
| SES-S | School-related self-efficacy scale | Jerusalem, Schwarzer 1999 | Gouda *et al.* 2016 |
| SRS | Self-regulation scale | Schwarzer, Jerusalem 2008 | Luong *et al*. 2019 |
|  | Short compulsive internet use scale | Besser, Rumpf 2017 | Guertler *et. al.* 2025 |
| Single-item measure of stress | Single-item measure of stress symptoms | Elo *et al.* 2003 | Guertler *et al.* 2025 |
| SSL | Student self-report on social and learning behaviour | Petermann, Petermann 2014 | Vasileva *et al.* 2019 |
|  | Subjective happiness scale | Lyubomirsky, Lepper 1999 | Schiepe-Tiska et al. 2015 |
|  | Swiss juvenile study | Jacobs Foundation 2015 | Haug *et al.* 2021 |
| LSL | Teacher report on social and learning behaviour | Petermann, Petermann 2013 | Vasileva *et al.* 2019 |
| TAI-E | Test anxiety inventory – emotionality | Schwarzer, Jerusalem 1999 | Gouda *et al.* 2016 |
| TAI-W | Test anxiety inventory – worry | Schwarzer, Jerusalem 1999 | Gouda *et al.* 2016 |
| TCT-DP | Test for creative thinking – drawing production | Urban & Jellen (1995) | Gouda *et al.* 2016, Luong *et al.* 2019 |
| UWES | Utrecht work engagement scale | Schaufeli et al. 2002 | Gouda *et al.* 2016 |
| VCT | Verbal creativity test | Various | Luong et al. 2019 |
| WHO-5 | Who-5 well-being index | WHO 1998 | Haug *et al.* 2021 |
| YSR | Youth self-report, German version | Achenbach 1991, Arbeitsgruppe deutsche child behavior checklist 1998 | Eppelmann *et al*. 2018 |

**Supplementary File 5.** Harvest plot coding table

| Study | Label | Area | Construct | Score | Mixed | Design | Mode | School | Quality |
| --- | --- | --- | --- | --- | --- | --- | --- | --- | --- |
| Eppelmann et al. 2018 | SBT | SBT | Self-management | 10 | TRUE | cRCT | P | G | M |
| Eppelmann et al. 2018 | SBT | SBT | Health-related outcomes | 0 | FALSE | cRCT | P | G | M |
| Fichtner et al. 2023 | LQEH | LQEH | Self-awareness | 10 | FALSE | QED | P | M | W |
| Fichtner et al. 2023 | LQEH | LQEH | Social awareness | 10 | FALSE | QED | P | M | W |
| Fichtner et al. 2023 | LQEH | LQEH | Relationship skills | 1 | TRUE | QED | P | M | W |
| Frenkel et al. 2020 | 8-sam | 8-sam | Self-awareness | 10 | TRUE | cRCT | P | G | M |
| Frenkel et al. 2020 | 8-sam | 8-sam | Self-management | 0 | FALSE | cRCT | P | G | M |
| Frenkel et al. 2020 | 8-sam | 8-sam | Health-related outcomes | 1 | FALSE | cRCT | P | G | M |
| Gouda et al. 2016 | MBSR-G | MBSR-G | Self-awareness | 20 | TRUE | cRCT | P | G | W |
| Gouda et al. 2016 | MBSR-G | MBSR-G | Self-management | 20 | FALSE | cRCT | P | G | W |
| Gouda et al. 2016 | MBSR-G | MBSR-G | Relationship skills | 1 | TRUE | cRCT | P | G | W |
| Gouda et al. 2016 | MBSR-G | MBSR-G | Health-related outcomes | 2 | TRUE | cRCT | P | G | W |
| Guertler et al. 2025 | R4L | R4L | Relationship skills | 1 | FALSE | cRCT | D | M | M |
| Guertler et al. 2025 | R4L | R4L | Health-related outcomes | 1 | TRUE | cRCT | D | M | M |
| Haug et al. 2021 | SC | SC | Relationship skills | 1 | FALSE | cRCT | D | U | M |
| Haug et al. 2021 | SC | SC | Health-related outcomes | 1 | TRUE | cRCT | D | U | M |
| Lang et al. 2016 | EPHECT | EPHECT | Self-management | 10 | TRUE | cRCT | P | V | M |
| Lang et al. 2016 | EPHECT | EPHECT | Health-related outcomes | 1 | FALSE | cRCT | P | V | M |
| Luong et al. 2019 | MBSR-L | MBSR-L | Self-awareness | 20 | TRUE | QED | P | G | W |
| Luong et al. 2019 | MBSR-L | MBSR-L | Self-management | 20 | FALSE | QED | P | G | W |
| Luong et al. 2019 | MBSR-L | MBSR-L | Relationship skills | 1 | TRUE | QED | P | G | W |
| Luong et al. 2019 | MBSR-L | MBSR-L | Health-related outcomes | 2 | FALSE | QED | P | G | W |
| IFT 2023 | MZO | MZO | Self-awareness | 0 | FALSE | cRCT | D | V | M |
| IFT 2023 | MZO | MZO | Responsible decision-making | 2 | FALSE | cRCT | D | V | M |
| IFT. 2023 | MZO | MZO | Health-related outcomes | 1 | TRUE | cRCT | D | V | M |
| Schiepe-Tiska et al. 2015 | SG | SG | Self-awareness | 10 | TRUE | QED | P | V | W |
| Schiepe-Tiska et al. 2015 | SG | SG | Health-related outcomes | 1 | TRUE | QED | P | V | W |
| Vasileva et al. 2019 | Jobfit | Jobfit | Self-awareness | 20 | FALSE | QED | P | M | M |
| Vasileva et al. 2019 | Jobfit | Jobfit | Self-management | 10 | TRUE | QED | P | M | M |
| Vasileva et al. 2019 | Jobfit | Jobfit | Social awareness | 10 | TRUE | QED | P | M | M |
| Vasileva et al. 2019 | Jobfit | Jobfit | Relationship skills | 2 | FALSE | QED | P | M | M |
